# A Robust GWSS Method to Simultaneously Detect Rare and Common Variants for Complex Disease

**DOI:** 10.1371/journal.pone.0120873

**Published:** 2015-04-16

**Authors:** Chung-Feng Kao, Jia-Rou Liu, Hung Hung, Po-Hsiu Kuo

**Affiliations:** 1 Department of Public Health, Institute of Epidemiology and Preventive Medicine, National Taiwan University, Taipei, Taiwan; 2 Department of Public Health, Chang Gung University, Taoyuan,Taiwan; 3 Research Center for Genes, Environment and Human Health, National Taiwan University, Taipei, Taiwan; McMaster University, CANADA

## Abstract

The rapid advances in sequencing technologies and the resulting next-generation sequencing data provide the opportunity to detect disease-associated variants with a better solution, in particular for low-frequency variants. Although both common and rare variants might exert their independent effects on the risk for the trait of interest, previous methods to detect the association effects rarely consider them simultaneously. We proposed a class of test statistics, the generalized weighted-sum statistic (GWSS), to detect disease associations in the presence of common and rare variants with a case-control study design. Information of rare variants was aggregated using a weighted sum method, while signal directions and strength of the variants were considered at the same time. Permutations were performed to obtain the empirical *p*-values of the test statistics. Our simulation showed that, compared to the existing methods, the GWSS method had better performance in most of the scenarios. The GWSS (in particular VDWSS-*t*) method is particularly robust for opposite association directions, association strength, and varying distributions of minor-allele frequencies. It is therefore promising for detecting disease-associated loci. For empirical data application, we also applied our GWSS method to the Genetic Analysis Workshop 17 data, and the results were consistent with the simulation, suggesting good performance of our method. As re-sequencing studies become more popular to identify putative disease loci, we recommend the use of this newly developed GWSS to detect associations with both common and rare variants.

## Introduction

The search for common variants (CVs) that are reproducibly associated with complex human diseases has benefited from large-scale genome-wide association (GWA) studies. However, previously identified CVs only account for a small proportion of the trait variation [1,2], i.e. missing heritability [1]. Even with meta-analyses to combine large GWA datasets, similar situations occur [2]. It is suggested that a certain proportion of the missing heritability might come from rare variants (RVs), which have low frequency in the population to increase the risk of developing complex diseases [3–5]. Unfortunately, RVs are poorly covered in GWA studies [1], and the power of detecting their individual effects is low even in large scale studies [6]. A larger sample size, say more than 10,000 subjects, is required to detect disease-associated variants with minor-allele frequency (MAF) less than 0.01 [7]. Recently, there is increasing evidence in support of the contributions of RVs to the risk of diseases, such as *CLEC16A* gene for type-1 diabetes and *ANGPTL* gene for metabolism traits [8,9]. With the advancement of high-throughput sequencing technologies, large amounts of sequencing data would be rapidly produced in the future years, which provides a unique opportunity to detect disease associated variants, in particular for RVs.

There are two main hypotheses underlying complex human diseases, the common-disease common-variant (CDCV) and the common-disease rare-variant (CDRV), and they are not mutually exclusive in many cases. The combination of CVs and RVs may exert their effects through synthetic association (i.e., synergetic effect) or have independent effects on the trait [10]. For instance, Ionita-Laza *et al* [11] uncovered six CVs (*p*-value = 0.03~1×10^−4^) and one RV (*p*-value = 1.7×10^−7^) in *NOD2* that affect the risk of developing Crohn disease. Wessel *et al* [12] identified both common and rare variants at *CHRNB2* and *CHRNA5* to exhibit significant associations with nicotine dependence. Nejentsev *et al* [13] found four RVs (*p*-value = 1.3×10^−3^~2.1×10^−16^) and one CV (*p*-value = 9.5×10^−17^) in the *IFIH1* gene to be protective against type-1 diabetes. In addition, one review article reported that both common and rare genetic variants in two genes (*LPL* and *APOA5*) play roles in the development of hypertriglyceridemia [14]. In prior statistical approaches to identify rare variants (which we describe below), very few of them deal with both common and rare genetic variants. Since both types of genetic markers in the same gene may exert their effects for disease traits, we propose a new method in the present study to identify common variants as well as rare variants for complex diseases.

In the last decade, several association tests have been developed for identifying RVs [9,11,15–25]. These methods may have limited power to detect the true signals under certain conditions. In particular, various collapsing and weighting schemes have impacts on the performance of association testing as described below. Firstly, some test methods, including weighted sum-test (WSS) [19], variable-threshold (VT) [23], and *w*SSU [15], sum over RVs where rare information were weighted by MAFs. Under this condition, a rarer (common) variant is always corresponding to a larger (smaller) weight. Moreover, these methods and kernel-based adaptive cluster (KBAC) [9], Sum Test [22] and the combined multivariate and collapsing (CMC) [17] do not take association directions into account. This could result in power loss in detecting associations, especially in the presence of CVs and/or in the presence of both harmful and protective influences. Secondly, methods that adopt a weighting scheme based on MAF, such as WSS [19], VT [23], sequence kernel association test (SKAT), where two types of weighting schemes (the beta density weight function, SKAT_b_; and the equal weigh function, SKAT_1_) are suggested [24], the optimal test of the SKAT (SKAT-O) [25], and *w*SSU [15], may misclassify signal CVs as noise variants (or *vise versa*), during the collapsing procedure. In addition, the rarer allele frequencies do not always imply the higher associations. Thus, these methods might be sometimes difficult to identify a signal RV, in particular when the RV is even rarer in normal population than in affected samples [26]. We noticed that Ionita-Laza et al [11] further modified the SKAT [24] to detect both CVs and RVs, which considered the weighted sum as the test statistic (named SKAT-A and SKAT-C). The SKAT-A and SKAT-C combine SKAT tests with adaptive sum test [27] and sum test [22], respectively, for the joint effects of RVs and CVs. Thirdly, methods that adopt a weighting scheme using odds ratio (OR), such as OR weighted sum test (ORWSS) [16], often suffer from the problem of unstable estimation due to sparse or empty cells in the tables for estimating OR for genetic effects, even after the adjustment in adding 0.5 to each cell of contingency table, especially when only RVs are present (this phenomenon is also observed in our simulation results). Finally, KBAC [9] clusters variants together based on combinations of genotypes. However, the number of combinations will be largely increased if too many variants are present, and it is difficult to specify which combinations are causal.

In this study, we reviewed recently proposed methods for the detection of disease associations for both CVs and RVs for comparisons. To overcome aforementioned methodological limitations, we also proposed a class of new methods based on *t*-statistic, to account for association directions, the magnitude of association, and the presence of noise variants. In addition, we utilized a “filtering algorithm” with or without a threshold to collapse information of rare variants collectively in detecting disease associated variants. In the cases where rare and common variants altogether contribute to the etiology of complex traits, our new methods had higher power in detecting signal variants. We conducted a series of simulations to evaluate detection power in different scenarios, including linkage disequilibrium (LD) structure among variants, association directions, MAF distribution of RVs, and the ratio of signal and noise variants. In addition, we applied our method to the Genetic Analysis Workshop 17 (GAW17) dataset to evaluate the performance of our method.

## Methods

### The GWSS Method

One major aim of this research is to propose a robust test method to overcome the difficulties encountered by existing methods. In view of the robustness, we aim to extend the idea of WSS without specifying any distributional assumption, which includes two core components. First, a weighted sum of RVs is used to aggregate the rare information. Commonly used weights include MAF and OR, but they will suffer the problem of low power as mentioned in the previous section. Second, with the aggregated variants, a summary measure is adopted to obtain the final genetic scores and the corresponding *p*-values. From these two considerations, we propose a generalized weighted sum statistic (GWSS), which includes various existing methods as special cases. Note that most of existing methods need a predetermined value for MAF to define “rare variants”. We believe that a data-driven approach for defining rare variants is more suitable, especially in the presence of signal CVs. We thus also incorporate the idea of VT [23] into GWSS (see Step 3 of the GWSS algorithm below). It is the data-driven weighting scheme that makes GWSS possible to simultaneously consider the effects of CVs and RVs. To formally describe GWSS, we consider a case-control study that examines *k* variants with *n* subjects (*n*_1_ cases and *n*_0_ controls). For subject *i*, let *G*_*ij*_ ∊ {0,1,2} be the number of minor allele in variant *j* and *D*_*i*_ ∊ {1,0} be the disease status (yes/no). For variant *j*, let *q*_*j*_ be its adjusted MAF [19] in the control group, and let *OR*_*j*_ be its estimated odds ratio. The algorithm of GWSS is described below.

### GWSS Algorithm:

1. Given a threshold *θ* of MAF, obtaining the aggregated genotype for each subject *i* as$G_{i}(\theta )=\sum_{j=1}^{k}\{w_{j}\cdot I(q_{j}\leq \theta )\}G_{ij}$, where *w*_*j*_ is one of the following four possible weights:
  - $w_{j}^{MAF}=\frac{1}{\sqrt{n_{0}q_{j}(1-q_{j})}}$
  - $w_{j}^{OR}=\log(OR_{j})$
  - $w_{j}^{D}=sign(w_{j}^{OR})=I(OR_{j}>1)-I(OR_{j}<1)$
  - $w_{j}^{DW}=w_{j}^{D}\times w_{j}^{MAF}=\frac{\left\{I(OR_{j}>1)-I(OR_{j}<1)\right\}}{\sqrt{n_{0}q_{j}(1-q_{j})}}$
2. Based on${\left\{G_{i}(\theta ),D_{i}\right\}}_{i=1}^{n}$, calculate the summary statistic *S*_*θ*_ using one of the following two summary measures:
  - rank-sum:$\sum_{i∊\left\{D_{i}=1\right\}}rank\left\{G_{i}(\theta )\right\}$
  - *t*-sum: $\frac{\left|mean\left\{G_{i}(\theta )|D_{i}=1\right\}-mean\left\{G_{i}(\theta )|D_{i}=0\right\}\right|}{\sqrt{Var\left\{G_{i}(\theta )|D_{i}=1\right\}/n_{1}+Var\left\{G_{i}(\theta )|D_{i}=0\right\}/n_{0}}}$
3. If *θ* is known a priori, define *S* = *S*_*θ*_. Otherwise, repeat Steps 1–2 for all *θ* ∊ Θ and calculate *S* = sup_*θ*∊Θ_ *S*_*θ*_ as the summary statistic, where Θ is a predetermined set of possible values of *θ*.
4. Obtain the empirical *p*-value of *S* via randomly permuting the disease status, while keeping the case/control ratio constant.

The robustness nature of GWSS is threefold as described below:

1. The weight$w_{j}^{MAF}$, which has been used in constructing WSS [19], implicitly assumes that RVs are more likely to be associated with disease. However, $w_{j}^{MAF}$is limited to detect only harmful association but ignores the protective one. Further, Feng *et al*. [16] proposed ORWSS, which used the weight $w_{j}^{OR}$ to estimate association strength and directions simultaneously. Unfortunately, $w_{j}^{OR}$suffers the problem of instability in estimating the association strength of RVs. It therefore motivates us to propose the weight$w_{j}^{D}$, which is a special case of aSum when setting the cutoff as 1 [27,28], that considers the direction of OR but ignores the information of strength. The information of strength, however, does play a role in affecting the testing performance. In view of this point, instead of using the unstably estimated OR directly, we propose the weight $w_{j}^{DW}$ that incorporates the information of strength in a robust manner. To see this, we prove that |OR-1| and |log(OR)| are both decreasing functions of the MAF of the control group under the assumption of rare disease (see S1 Appendix for the proof). This result implies that $w_{j}^{MAF}$ can be used as a surrogate for the association strength. As a result, the weight $w_{j}^{DW}=w_{j}^{D}\times w_{j}^{MAF}$ provides a more robust estimation in both association strength (i.e.,$w_{j}^{MAF}$) and direction (i.e.,$w_{j}^{D}$), and is able to detect the signal variants with both harmful and protective associations. We note that any weight function can be used in our GWSS. For example, we can also use the beta density weight function of SKAT [24] as *w*_*j*_.
2. In Step 2, based on the aggregated genotype *G*_*i*_(*θ*) for each subject *i*, two summary measures of${\left\{G_{i}(\theta )\right\}}_{i=1}^{n}$, the “rank-sum” and “*t*-sum”, are considered. The “rank-sum” statistic, which borrows the idea of the Wilcoxon rank-sum statistic, is robust against model assumptions, but at the cost of being less efficient when the distributional assumption is valid. Thus, when the sample size is moderate (i.e., the normal assumption is approximately hold), we suggest to use the more efficient “*t*-sum” to summarize information.
3. Before implementing WSS, a threshold *θ* for defining RVs should be determined. The same idea is also used in GWSS, which involves the indicator function *I*(*q*_*j*_ ≤ *θ*) in Step 1 of GWSS algorithm. This indicator function, however, ignores all variants with MAF >*θ*, which will lose detection power in the presence of signal CVs. Needless to say, it is also hardly the case that researchers have prior knowledge about the value of such *θ*. It turns out that a data-driven approach for determining *θ* is preferable. We thus follow the idea of VT to use the maximum value of *S*_*θ*_ among possible values of *θ* (see Step 3) as the summary statistic, which is able to adapt to various situations of signal/noise RVs and CVs (i.e., is more robust than using a predetermined threshold *θ*).

We close this section by noting that GWSS includes many existing test methods as special cases, such as WSS [19] and ORWSS [16]. See Table 1 for the details. Comparing with WSS and ORWSS, “D” denotes the method using $w_{j}^{D}$ and “DW” denotes the method using $w_{j}^{DW}$ in Step 1, “*t*” denotes the method using *t*-sum in Step 2, and “V” denotes the method that taking maximum over *θ* in Step 3.

**Table 1:** Various test methods of the generalized weighted sum statistic (GWSS).

Method	Weight	Summarized Scheme	Summary Statistic
	(Step 1)	(Step 2)	(Step 3)
WSS	$w_{j}^{MAF}$	rank-sum	*S*_*θ*_
ORWSS	$w_{j}^{OR}$	rank-sum	*S*_*θ*_
WSS-*t*	$w_{j}^{MAF}$	*t*-sum	*S*_*θ*_
ORWSS-*t*	$w_{j}^{OR}$	*t*-sum	*S*_*θ*_
DSS-*t*	$w_{j}^{D}$	*t*-sum	*S*_*θ*_
DWSS-*t*	$w_{j}^{DW}$	*t*-sum	*S*_*θ*_
VWSS-*t*	$w_{j}^{MAF}$	*t*-sum	*S* = sup_*θ*_ *S*_*θ*_
VORWSS-*t*	$w_{j}^{OR}$	*t*-sum	*S* = sup_*θ*_ *S*_*θ*_
VDSS-*t*	$w_{j}^{D}$	*t*-sum	*S* = sup_*θ*_ *S*_*θ*_
VDWSS-*t*	$w_{j}^{DW}$	*t*-sum	*S* = sup_*θ*_ *S*_*θ*_

## Simulation Studies

### Simulation Settings

We conducted simulation studies to compare the performance of the proposed GWSS with existing methods. Assume that a study examines *k* SNPs with 500 cases and 500 controls. Two settings for MAF (denoted by *MAF*_*j*_) of RV were considered. For the case of identical distribution, signal and noise RVs were both generated from Uniform(0.001, 0.01). For the case of different distributions, signal and noise RVs were generated from Uniform(0.001, 0.005) and Uniform(0.001, 0.01), respectively, i.e. the MAFs of signal variants are lower than noise variants [28]. CVs are generated from Uniform(0.01, 0.1) in both cases. Conditional on *MAF*_*j*_, let the latent vector $Z_{i}^{\left(l\right)}={\left(Z_{i1}^{\left(l\right)},\ldots ,Z_{ik}^{\left(l\right)}\right)}^{T},l=1,2,$ be independent and follow a multivariate normal distribution with mean zero and $Cov(Z_{iu}^{\left(l\right)},Z_{iv}^{\left(l\right)})=\rho^{\left|u-v\right|}$, and define$G_{ij}=\sum_{l=1}^{2}I\left\{\;Z_{ij}^{\left(l\right)}\leq \Phi^{-1}\left(MAF_{j}\right)\;\right\}$, where ρ is a LD measure index [29] and Φ(⋅) represents the cumulative distribution function of standard normal. The positions of signal/noise RVs/CVs are also randomly specified (the same assumption was adopted in Basu & Pan, 2011). Finally, the disease status *D*_*i*_ is generated from the logistic regression model

$$Logit\left\{\mathrm{Pr}(D_{i}=1|G)\right\}=Logit\left(0.05\right)+\left(\sum_{j=1}^{4}\log(OR_{j})\times G_{ij}\right)+\log(3)\times I\left(\sum_{j=5}^{8}G_{ij}>0\right).$$

This regression model is designed so that the background disease prevalence is 0.05 with 8 signal variants and *k* − 8 noise variants. The effects of SNP 1–4 are additive, and the effects of SNP 5–8 are non-additive. As to SNP 5–8, the effect is 3 if any of the SNP 5–8 has mutation. We note that the non-additive effect of multi-markers may be jointly incorporated in haplotypes [30] to capture the combined effects of variants signals, and this setting is to reflect a situation where no specific mutation is necessary but any of them is sufficient to increase the risk of disease. We consider different combinations of the following situations: (1) *ρ* = 0 or 0.7 for LD between markers; (2) *OR*_*j*_ for (RVs, CVs) is (2, 1.5) or (1/2, 1/1.5); (3) $\left(n_{true}^{RV},\;n_{true}^{CV},\;n_{noise}^{RV},\;n_{noise}^{CV}\right)=$(8, 0, 0, 0), (8, 0, 8, 0), (8, 0, 4, 4), (7, 1, 8, 0) and (7, 1, 4, 4), where$n_{true}^{RV}$, $n_{true}^{CV}$, $n_{noise}^{RV}$ and $n_{noise}^{CV}$ represents the number of signal RVs, signal CVs, noise RVs, and noise CVs, respectively, and$k=n_{true}^{RV}+n_{true}^{CV}+n_{noise}^{RV}+n_{noise}^{CV}$; and (4) same or different MAF distribution for signal and noise RVs. For all methods, the empirical *p*-values are calculated based on 500 permutations. The detection power is calculated as the proportion of test statistic attained significance level of 0.05 over 1000 simulation datasets.

## Results

S1 Table shows that the type I errors of all methods under each setting are controlled at the level of 0.05, except for the CMC method. Thus, we used the empirical *p*-value for CMC statistic instead in the following simulation. We only present methods with better performance in the Tables, including five existing methods (SSU*w*, *w*SSU, SKAT_b_, SKAT-C, SKAT-A) and five tests of our GWSS method (ORWSS-*t*, DWSS-*t*, VORWSS-*t*, VDWSS-*t* and VDSS-*t*). The results of comparisons with other methods are listed in the Supporting Tables. Table 2 and S2 Table demonstrate detection power for identical MAF distributions of signal and noise RVs. Details are described below under different scenarios.

**Table 2:** Detection power for identical MAF distributions of signal and noise rare variants (only list methods with better performance).

	*ρ =* 0										*ρ* = 0.7									
	(a) *OR*_*j*_ = 2 (RVs), 1.5 (CV)					(b) *OR*_*j*_ = 1/2 (RVs), 1/1.5 (CV)					(c) *OR*_*j*_ = 2 (RVs), 1.5 (CV)					(d) *OR*_*j*_ = 1/2 (RVs), 1/1.5 (CV)				
$n_{true}^{RV}$	8	8	8	7	7	8	8	8	7	7	8	8	8	7	7	8	8	8	7	7
$n_{true}^{CV}$	0	0	0	1	1	0	0	0	1	1	0	0	0	1	1	0	0	0	1	1
$n_{noise}^{RV}$	0	8	4	8	4	0	8	4	8	4	0	8	4	8	4	0	8	4	8	4
$n_{noise}^{CV}$	0	0	4	0	4	0	0	4	0	4	0	0	4	0	4	0	0	4	0	4
SSU*w*	0.96	0.88	0.88	0.90	0.91	0.92	0.83	0.85	0.88	0.86	1.00	0.98	0.98	0.99	0.99	0.71	0.69	0.71	0.72	0.74
*w*SSU	0.98	0.94	0.95	0.95	0.95	0.91	0.81	0.86	0.87	0.88	1.00	0.99	1.00	0.99	0.99	0.70	0.70	0.76	0.74	0.76
SKAT_b_	0.96	0.92	0.91	0.93	0.95	0.95	0.89	0.89	0.94	0.94	1.00	0.99	0.99	0.99	0.99	0.80	0.78	0.78	0.82	0.79
SKAT-C	0.96	0.92	0.84	0.93	0.93	0.94	0.89	0.81	0.92	0.89	1.00	0.99	0.99	0.99	0.98	0.80	0.78	0.65	0.79	0.73
SKAT-A	0.96	0.92	0.89	0.91	0.93	0.94	0.89	0.86	0.93	0.91	1.00	0.99	0.98	0.98	0.99	0.80	0.78	0.70	0.81	0.75
ORWSS-*t*	0.97	0.91	0.89	0.92	0.92	0.93	0.86	0.87	0.90	0.89	0.99	0.94	0.94	0.97	0.96	0.80	0.75	0.73	0.81	0.78
DWSS-*t*	0.97	0.93	0.92	0.93	0.93	0.94	0.85	0.85	0.89	0.88	0.99	0.96	0.95	0.97	0.96	0.80	0.71	0.71	0.76	0.75
VORWSS-*t*	0.97	0.91	0.89	0.92	0.92	0.93	0.86	0.87	0.90	0.89	0.99	0.94	0.95	0.97	0.97	0.80	0.74	0.74	0.81	0.81
VDWSS-*t*	0.98	0.93	0.93	0.93	0.93	0.94	0.85	0.88	0.89	0.89	0.99	0.96	0.97	0.97	0.97	0.80	0.72	0.74	0.77	0.78
VDSS-*t*	0.97	0.93	0.92	0.93	0.92	0.94	0.88	0.88	0.92	0.90	1.00	0.96	0.97	0.98	0.97	0.81	0.75	0.76	0.81	0.76

### Harmful Association Direction Without LD (ρ = 0)

In Table 2 and S2 Table (column (a)), when there are only RVs, detection powers of almost all methods are larger than 95%. Among methods, Sum test, KBAC, WSS and WSS-*t* have the best performance. When there are noise variants, MAF-based methods such as *w*SSU, VT, WSS-*t*, and VWSS-*t* have better performances than others, since the noise CVs are down-weighted by the value of MAF in these methods. In contrast, the detection powers of SSU, Sum Test, KMR, C-alpha, SKAT_1_ (SKAT with equal weight) and DSS-*t* decrease dramatically, suggesting that some underlying assumptions in these methods are inappropriate in the presence of noise CVs. Comparing WSS with WSS-*t* for which one uses “rank-sum” and the other uses “*t*-sum”, WSS-*t* has better performance than WSS, especially when noise CVs are present. The same pattern exists between ORWSS and ORWSS-*t*. This observation suggests that, in the presence of noise CVs and with moderate sample size, using “*t*-sum" to obtain the summary score is able to extract more information and, hence, is more efficient than using “rank-sum”.

As mentioned in the Introduction section, OR provides information of both association strength and directions, but OR-based weighting scheme might result in loss of power due to the unstable estimation of association strength with sparse data in tabulated genotypic data (i.e. a contingency table). Taking two special cases of GWSS, ORWSS-*t* (using the weight$w_{j}^{OR}$) and DSS-*t* (using the weight$sign(w_{j}^{OR})$) to exemplify. When there are only RVs involved, we observe that DSS-*t* suffices to have better performance than ORWSS-*t*. On the other hand, when noise CVs exist, ORWSS-*t* and DWSS-*t* have better performances than DSS-*t*, indicating that the strength of association can assist the detection process when it can be estimated well. Moreover, we can observe that VDSS-*t* has a better performance than DSS-*t* in the presence of CVs. It indicates that, whether the CVs exist or not, the VT approach is able to improve the detection power even we do not estimate the association strength directly.

### Opposite Association Directions Without LD (ρ = 0)

Simulation results are provided in Table 2 and S2 Table (column (b)). It can be seen that variants protectively associated with disease can be detected by SSU, KBAC, KMR, C-alpha, and SKAT-type methods (including SKAT_1_, SKAT_b_, SKAT-C and SKAT-A) if there is no noise CV. In the presence of noise CVs, SSU*w* (assuming the variance of each variant does not equal to one), *w*SSU (assigning small weight to noise CVs), SKAT_b_ (SKAT using beta(1,25) density function evaluated at *q*_*j*_ as the weight) and SKAT-C/A (using an optimal weight for combining CV and RV test statistics) perform better as expected. As to the class of GWSS methods, ORWSS-*t*, DWSS-*t*, VORWSS-*t*, VDWSS-*t* and VDSS-*t* perform well uniformly in all situations. These results reveal that using OR-based weighting ($w_{j}^{OR}$,$w_{j}^{DW}$) is able to detect associations for both harmful and protective effects. Besides, when the weight itself has the ability to identify association directions, the corresponding VT-version methods (e.g. VORWSS-*t*, VDWSS-*t*, and VDSS-*t*) still have improved performances. In contrast, we observe power losses in Sum Test, CMC-p (cutoff-point is fixed at 0.01), WSS-*t* and VWSS-*t* when opposite association directions are present. The failure to detect associations using Sum Test and CMC-p is due to the offset of harmful and protective effects during the summation process. The power losses in WSS-*t* and VWSS-*t* are because the weight $w_{j}^{MAF}$ has no ability to assist identifying protective associations. Comparing with WSS-*t*, WSS using rank-sum is less affected by the presence of RVs with opposite directions and has a higher detection power than using “*t*-sum”, which is a benefit of using the more robust “rank-sum” summary measure.

### With LD (ρ = 0.7)

When variants are correlated with each other (see Table 2 and S2 Table columns (c), (d)), the results are similar to the scenarios of *ρ* = 0: (1) SSU, KMR, C-alpha, ORWSS and SKAT_1_ performed worse in the presence of CVs; (2) for GWSS, using *t*-sum is better than using rank-sum in most of the situations; (3) methods using OR-based weighting scheme can identify protective association while methods using MAF-based weighting scheme cannot; (4) the VT-version of GWSS methods are superior to non-VT-version ones whether the CVs and protective association exists or not. The impact of LD among variants depends heavily on the association directions among variants. When the effects of signal variants are in the same direction, the joint signals become stronger than the case of uncorrelated variants. In this situation, it is easier to detect association and the detection power is much higher than the case of *ρ* = 0 (Table 2 and S2 Table column (c)). On the other hand, when both harmful and protective variants exist simultaneously, the signals become weaker and are not easy to be detected (Table 2 and S2 Table column (d)), due to the offset of opposite association effects.

### Different MAF Distributions of Signal/Noise RVs

**Fig 1:**
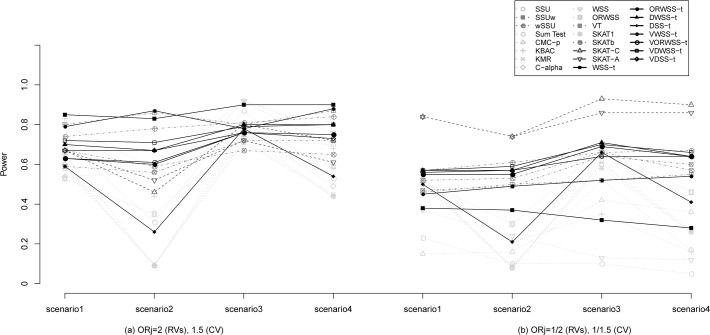
Detection power (ρ = 0) for different MAF distributions of signal and noise rare variants. The left panel considers OR_j_ = 2 for rare variants (RVs) and 1.5 for common variant (CV), and the right panel considers OR_j_ = 1/2 for RVs and 1/1.5 for CV. Each panel considers four scenarios: scenario1 considers 8 signal RVs and 8 noise RVs; scenario2 considers 8 signal RVs, 4 noise RVs and 4 noise CVs; scenario3 considers 7 signal RVs, 1 signal CV and 8 noise RVs; and scenario4 considers 7 signal RVs, 1 signal CV, 4 noise RVs and 4 noise CVs. The results were based on 1000 subjects (500 cases and 500 controls). All empirical *p*-values were calculated from 500 permutations. The detection power is defined as the proportion of test statistic attained significant level 0.05 over 1000 simulations. CMC method use 0.01 as a threshold for rare variants. SKAT_1_ and SKAT_b_ represents SKAT using equal weight and using beta(1,25) density function evaluated at *q*_*j*_ as the weight, respectively. SKAT-C and SKAT-A combined SKAT and sum test and adaptive sum test, respectively.

S3 Table demonstrates the Type I errors and detection powers under different MAF distributions between signal and noise RVs. Comparing to the scenarios (Fig 1 and S3 Table) with identical MAF distribution (please see results in Table 2 and S2 Table), we found almost the same patterns in all methods. Moreover, we found that VT and WSS-*t* using MAF-based weighting scheme outperform other methods in OR>1 scenario (S3 Table column (a)), since the noise variants are down-weighted by MAF. Although both SKAT-C and SKAT-A perform worse than GWSS and *w*SSU in the scenarios of different MAF distribution across CVs and RVs with OR>1(S3 Table column (a)), they outperform other methods in OR<1 scenario (S3 Table column (b)). It reveals the non-robustness of SKAT-C/A, whose performances will be heavily affected by the varying situations. As expected, the detection powers of VT and WSS-*t* for detecting signal CVs with protective associations are low. On the other hand, the proposed DWSS-*t* and VDWSS-*t* take into account the association strength and direction as well as the MAF distributions of RVs, and are detected to perform well in all situations. Notably, in comparison with the scenarios of identical MAF distribution for both noise and signal RVs (see Table 2 and S2 Table columns (a), (b)), all methods encounter a dramatically decline in power (Fig 1 and S3 Table columns (a), (b)). One reason is that the MAF distributions of signal RVs in the scenario of different MAF distributions (i.e. MAF~Uniform(0.001,0.005)) are rarer than those in the scenario of identical MAF distributions (i.e. MAF~Uniform(0.001,0.01)), and thus, the signals are more difficult to be identified.

### Summary of Simulation Studies

We summarize the simulation results for the performances of all methods under different scenarios in Table 3 and S4 Table. The power differences across all methods in different scenarios are first calculated. We classify a method to be sensitive (●), slightly sensitive (Δ), or non-sensitive (X) if the difference is larger than 0.1, between 0.05 and 0.1, or less than 0.05, respectively. We found that the decline in power is mainly caused by opposite association directions and the presence of noise CVs, followed by the presence of noise RVs (Table 3 and S4 Table column (a)). We also evaluate the influences of two factors simultaneously (association directions and the existence of noise variants) in Table 3 and S4 Table column (b). For instance, KBAC is less sensitive in the presence of opposite directions and noise CVs, but it has worse performance when both factors exist simultaneously. Moreover, the MAF distributions of signal and noise variants have impacts on the detection power. For example, ORWSS and SKAT are robust to the presence of opposite directions for identical MAF distributions, but are not robust for different MAF distributions. It is also found that the performance of SKAT is sensitive to the selection of the weight function, since SKAT_1_ and SKAT_b_ have totally different behaviors. SKAT-C and SKAT-A are also sensitive to the existence of noise variants, MAF distributions, the association directions, and the chosen weight function. In general, the performances of DWSS-*t*, VDWSS-*t*, and *w*SSU are robust against the association directions and the presence/absence of signal /noise CVs.

**Table 3:** Robustness of all methods in situations of identical/different MAF distributions of signal and noise rare variants (only list methods with better performance).

	(a) One factor			(a) Two factors		
	Direction	Noise RVs	Noise CVs	Noise RVs X Direction	Noise CVs X Direction	signal CVs X Direction
SSU*w*	X	Δ	X	X	X	X
*w*SSU	Δ	X	X	Δ	X	X
SKAT_b_	X (●)	X (Δ)	X (Δ)	X	X	X
SKAT-C	X	X	●	X	X	X
SKAT-A	X	X	X (●)	X	X	X
ORWSS-*t*	X	Δ	X	X	X	X
DWSS-*t*	X	X	X	X	X	X
VORWSS-*t*	X	Δ	X	X	X	X
VDWSS-*t*	X	X	X	X	X	X
VDSS-*t*	X	X	X	X	X	X

It can be seen that *w*SSU and VDWSS-*t* have comparable performances, and are the best performers among all methods. We should emphasize that *w*SSU assumes the validity of a logistic regression model, and thus a good performance is expected under our simulation settings. On the other hand, VDWSS-*t* is totally nonparametric tests that does not require any model specification, and is robust to various situations of MAF and signal /noise variants. As a result, VDWSS-*t* is expected to be more applicable in practice since it is rarely the case that we know what the true model is.

## Application

To evaluate the performance of our GWSS method, we used the GAW17 mini-exome dataset (http://www.gaworkshop.org/gaw17, without true answers). The GAW17 dataset consists of 697 unrelated individuals, each of whom provided genotypes (in total 24,487 SNPs assigned to 3,205 genes) that were sequenced from the 1000 Genomes Project (http://www.1000genomes.org). The simulated phenotypes used binary disease status that was generated for each individual (Almasy et al., 2011). We calculated association p-values for disease affected status based on 1000 permutations. We compared our GWSS method to a few commonly implemented methods, including SSU*w*, WSS, ORWSS, VT, and SKAT-type methods (results are shown in Table 4 and S5 Table).

**Table 4:** Effects of disease liability using the Genetic Analysis Workshop 17 data (only list methods with better performance).

Chromosome	1	1	2	3	3	4	9	10	11	13	14	18	19	22
Gene symbol	*ADAM15*	*MSH4*	*ARL6IP2*	*EPHB1*	*TRIM42*	*FAM13A1*	*SHC3*	*FRMPD2*	*DGKZ*	*FLT1*	*NFKBIA*	*MBD1*	*GDF15*	*SUSD2*
no. of RVs	22	16	9	6	33	27	4	42	17	25	6	10	4	36
no. of CVs	8	4	4	2	6	7	1	8	5	10	2	2	6	9
***p*-value:**														
SSU*w*	0.002	0.005	0.015	**0.001**	0.003	0.007	0.015	0.005	**0.0005**	**0.0005**	0.009	0.009	0.009	0.003
*w*SSU	0.005	0.018	0.004	0.039	**0.001**	0.007	0.002	0.009	0.003	0.003	0.020	0.011	0.003	0.019
SKAT_b_	0.014	0.798	0.022	0.244	0.060	**0.0002**	0.237	0.008	0.001	0.172	0.014	0.016	0.007	0.023
SKAT-C	0.008	0.002	0.069	0.121	0.058	**<.0001**	**0.001**	**0.001**	**<.0001**	0.006	0.016	0.004	0.005	0.009
SKAT-A	0.008	0.009	0.211	0.123	0.039	0.002	0.003	**0.001**	0.010	0.036	0.071	0.005	0.027	0.025
GWSS														
WSS-*t*	**0.001**	0.249	**0.001**	0.625	0.004	0.003	0.006	**0.001**	**0.001**	**0.001**	**0.001**	0.015	0.005	**0.001**
ORWSS-*t*	0.006	0.002	0.020	**0.001**	0.052	0.059	**0.001**	0.009	0.241	0.008	0.065	0.004	0.014	0.179
DWSS-*t*	0.006	0.002	0.014	0.002	0.035	0.046	0.040	0.008	0.079	0.004	0.034	0.012	0.002	0.079
DSS-*t*	0.012	0.002	0.071	**0.001**	0.587	0.099	0.002	0.003	0.025	0.006	0.123	0.010	0.007	0.085
VWSS-*t*	**0.001**	0.415	**0.001**	0.619	**0.001**	**0.001**	0.006	0.091	0.002	0.002	**0.001**	**0.001**	**0.001**	0.002
VORWSS-*t*	0.008	**0.001**	0.018	0.002	0.069	0.099	0.003	0.017	0.278	0.018	0.068	0.005	0.019	0.150
VDWSS-*t*	0.006	0.003	0.008	0.003	0.026	0.038	0.034	0.008	0.112	0.002	0.034	0.010	0.005	0.098
VDSS-*t*	0.090	0.003	0.013	**0.001**	0.119	0.026	**0.001**	0.066	0.141	0.008	0.133	0.289	0.003	0.063

To conduct the association tests of the GAW17 data, only genes that (1) had significant *p*-value≤0.001 detected in at least one of the GWSS, SSU*w* and SKAT-C/A methods, and (2) consisted of at least one RV and CV were considered. We listed fourteen genes that showed significantly aggregated signals to the disease affected status in Table 4 and S5 Table. Eight genes (*ARL6IP2*, *TRIM42*, *SHC3*, *FRMPD2*, *NFKBIA*, *MBD1*, *GDF15* and *SUSD2*) had the smallest *p*-value detected by one of our GWSS method (i.e., WSS-*t*, ORWSS-*t*, VWSS-*t* and VDSS-*t*). The *p*-values of the other six genes (*ADAM15*, *MSH4*, *EPHB1*, *FAM13A1*, *DGKZ* and *FLT1*) were also ranked in the top three among all methods. We found that SKAT-type methods performed well (*p*-values≤0.001) in seven genes, but completely failed to detect signals for three genes (*APL6IP2*, *TRIM42* and *NFKBIA*). Notably, three genes (*ARL6IP2*, *NFKBIA* and *MBD1*) can only be detected by our WSS-*t* and VWSS-*t*. These results suggested that our GWSS method had good performance among all methods. Taken together, we concluded that our GWSS method robustly showed good performance under different scenarios.

## Discussion

Various statistical testing strategies have been developed for identifying associations between RVs and complex disease traits [9,15–19,21,23,24]. With noted limitations in existing methods, we developed a robust GWSS method to identify rare and common disease-related variants simultaneously. The proposed GWSS method works well under most of the simulated scenarios. Comparing with existing methods, GWSS can correctly control the type-I error rate and achieve high detection powers under harmful and/or protective association settings, when LD structures among variants are absent (i.e. ρ = 0) or present (i.e. ρ = 0.7) (Table 2 and S2 Table).

Many existing methods suffer from low to moderate power loss when noise variants (rare or common) are present. The presence of noise variants can dramatically decrease the detection powers and increase the false positive results, which further complicate the interpretations of the analysis results. Importantly, by incorporating the idea of VT, our GWSS method can separate signal variants from noise ones in a data-driven approach. In practice, noise may also be introduced from calling rare variants and using imputed genotypes [7]. Methods like our GWSS can help to combine information from both RVs and CVs and further provide a basis to enhance our understanding of complex trait genetics.

Power loss may also occur when using methods assuming the same effect direction of variants, since in reality both risk and protective variants are present [31]. It is easy to see that risk effects are neutralized by protective effects in Sum Test and CMC-p methods. Similarly, methods using MAF as a weighting scheme, such as VT, WSS-*t*, and VWSS-*t* ignore associations with protective effects (i.e. OR<1), also encounter power loss. Although the protective associations can be detected by using OR as the weighting scheme, the unstable estimation of OR arises another difficulty. The newly proposed VDWSS-*t* exhibits a more robust estimation in both association strength and direction, which are more applicable in many situations.

The power of association testing also depends on the pattern of LD among markers [32], since noise variants may be associated with a signal variant due to LD. High LD among RVs and CVs often makes it difficult to detect which variants are the true association signals, and causes loss in detection power. In particular, when high LD exists among RVs (including both harmful and protective variants), a huge loss in power is observed due to restricted signals [15]. This situation is also found in our simulation studies. A dramatic power loss can be found in opposite association directions when noise variants are additionally present (Table 2 and S2 Table column (d)). The comprehensive influences of LD structures among variants in RVs detection need further studies.

It is found that WSS-*t* and ORWSS-*t* performed better than WSS and ORWSS, suggesting that the use of “*t*-sum” is beneficial especially when CVs are present. One reason is that “*t*-sum” utilizes more information than “rank-sum”, through considering both the mean shift and variation. In particular, when only RVs are involved and all variants have harmful effects, most of the genotypes are common homozygotes (i.e. *G*_*ij*_ = 0) and the aggregated genotypes (i.e. *G*_*i*_(*θ*)) close to zero in most of the subjects, forming a right-skewed distribution. In this situation, “rank-sum” is more appropriate due to its robustness against the normality assumption. Similarly, when the assumption of the same association direction among variants was violated, both WSS and WSS-*t* had poor performance, while the rank-sum slightly improved the detection power. In contrast, when CVs are involved (i.e. fewer cells with zero value show in tables of genotyping data) with moderate sample size, the distribution of aggregated genotypes would approximately follow the normal distribution, so using “*t*-sum” is suitable.

Weighting scheme plays an important role in successful identification of signal variants. Inappropriately using weighting scheme may lead to misclassification of noise variants to signal variants. For example, when all RVs have true signals and all CVs are noise, methods using MAF-based weighting can efficiently separate true signals from noise variants, thus yielding gains in power. When signal and/or noise CVs are present, however, methods such as SSU, Sum Test, KMR, C-alpha, and KBAC without considering the effects of CVs will suffer from certain degree of power loss. The choice of the weight function is also critical to some methods. For instance, SKAT and VDWSS-*t* perform nearly equally well under identical MAF distribution (Table 2 and S2 Table), but SKAT is not a good performer under different MAF distribution (Fig 1). We should emphasize that our GWSS adopts a robust weight function that is able to adapt to various situations, which is more applicable in practice. Additionally, the calculation of GWSS is not time-consuming. In fact, GWSS without using varying threshold values has very similar computational time to WSS. For one simulation data used in our Simulation section, it costs about 2 minutes to obtain *p*-values. When a varying threshold method is implemented, the calculation can still be completed within 6 minutes.

Due to the nonparametric nature of GWSS, however, currently there is no simple method to adjust for the effects of potential covariates, except the stratification method where one can implement GWSS within the strata. It is of great interest to extend GWSS to accommodate to include potential covariates.

In conclusion, our results demonstrated that the newly proposed GWSS method is able to deal with variants with bidirectional effects and association strength successfully. The GWSS can aggregate information from both CVs and RVs automatically even there is no prior knowledge. The GWSS also takes into account different LD patterns, numbers of signal/noise variants, and association directions. The GWSS can be applied to genome-wide sequence data to assist with the identification of signals of both common and rare variants, and to enhance our understanding of complex trait genetics.

## Supporting Information

S1 Appendix (DOC)

S1 Table Type I error (significant level = 0.05). (DOC)

S2 Table Detection power for identical MAF distributions of signal and noise rare variants (other methods). (DOC)

S3 Table Type I error (significant level = 0.05) and detection power (ρ = 0) for different MAF distributions of signal and noise rare variants. (DOC)

S4 Table Robustness of all methods in situations of identical/different MAF distributions of signal and noise rare variants (other methods). (DOC)

S5 Table Effects of disease liability using the Genetic Analysis Workshop 17 data (other methods and details of the GWSS method). (DOC)
