# Supplementary material for: A Robust GWSS Method to Simultaneously Detect Rare and Common Variants for Complex Disease
Source: PLoS One. 2015 Apr 16;10(4):e0120873. doi: 10.1371/journal.pone.0120873 (PMC4399906; doi:10.1371/journal.pone.0120873)
Supplement: S1 Appendix — (DOC) [file pone.0120873.s001.doc]

# Appendix S1

**Proposition:** *Let $D\in\{1,0\}$ be the disease status (yes/no), $G\in\{0,1,2\}$ be the number of minor allele and , where $p$ is the MAF of the control group. Assume $P(D=1)\approx0$ (i.e. rare disease)*

(a) If the population attributable risk (PAR) is fixed, then $|OR-1|$ is a decreasing function of $p$.

(b) Under the multiplicative model, if the contribution of a risk factor to the overall genetic variation (GV) is fixed, then $\left|\log\left(OR\right)\right|$ is a decreasing function of $p$.

Proof.

(a) Let $RR=\frac{P(D=1|G\neq0)}{P(D=1|G=0)}$ be the relative risk of disease caused by at least one copy of minor allele (i.e. $G\neq0$). The population attributable risk can be expressed as

$PAR:=\frac{P(D=1)-P(D=1|G=0)}{P(D=1)}=\frac{P(G\neq0)\cdot(RR-1)}{1+P(G\neq0)\cdot(RR-1)}$.

This implies

$|RR-1| = \left|\frac{PAR}{1-PAR}\cdot\frac{1}{P(G\neq0)}\right|$.

For rare disease, we have $RR\approx OR$ and $P(G\neq0)\approx P(G\neq0|D=0)$. Thus,

$|OR-1| \approx\left|\frac{PAR}{1-PAR}\cdot\frac{1}{P(G\neq0|D=0)}\right| \propto\text{ }\frac{1}{P(G\neq0|D=0)}$.

Note that where $P(G\neq0|D=0)=1-P(G=0|D=0)=1-(1-p)^{2}$ is an increasing function of $p$, which completes the proof.

(b) From Witte et al , the contribution of a risk factor to the overall genetic variation can be expressed as

$$GV=2p(1-p)\cdot\left|\log\left(RR\right)\right|^{2}$$

Thus, when GV is fixed, it can be seen that $\left|\log\left(RR\right)\right|^{2}$ is a decreasing function of $p$ on [0,0.5]. The proof is completed by noting that $RR\approx OR$ for rare disease.

Reference:

1. Witte JS, Visscher PM, Wray NR (2014) The contribution of genetic variants to disease depends on the ruler. Nature Reviews Genetics 15: 765-776.
