# Supplementary material for: A Robust GWSS Method to Simultaneously Detect Rare and Common Variants for Complex Disease
Source: PLoS One. 2015 Apr 16;10(4):e0120873. doi: 10.1371/journal.pone.0120873 (PMC4399906; doi:10.1371/journal.pone.0120873)
Supplement: S1 Table — (DOC) [file pone.0120873.s002.doc]

**Table S1. Type I error** **(significant level=0.05).**

|  | $\rho=0$ | | | | |  | $\rho=0.7$ | | | | |
| --- | --- | --- | --- | --- | --- | --- | --- | --- | --- | --- | --- |
| $n_{true}^{RV}$ | 8 | 8 | 8 | 7 | 7 |  | 8 | 8 | 8 | 7 | 7 |
| $n_{true}^{CV}$ | 0 | 0 | 0 | 1 | 1 |  | 0 | 0 | 0 | 1 | 1 |
| $n_{noise}^{RV}$ | 0 | 8 | 4 | 8 | 4 |  | 0 | 8 | 4 | 8 | 4 |
| $n_{noise}^{CV}$ | 0 | 0 | 4 | 0 | 4 |  | 0 | 0 | 4 | 0 | 4 |
| SSU | 0.039 | 0.048 | 0.043 | 0.051 | 0.050 |  | 0.052 | 0.037 | 0.051 | 0.049 | 0.050 |
| SSU*w* | 0.031 | 0.037 | 0.054 | 0.036 | 0.040 |  | 0.032 | 0.035 | 0.046 | 0.039 | 0.033 |
| *w*SSU | 0.040 | 0.057 | 0.055 | 0.064 | 0.048 |  | 0.052 | 0.042 | 0.047 | 0.069 | 0.038 |
| Sum Test | 0.042 | 0.046 | 0.063 | 0.043 | 0.045 |  | 0.052 | 0.058 | 0.042 | 0.053 | 0.049 |
| CMC | 0.076 | 0.106 | 0.076 | 0.098 | 0.057 |  | 0.077 | 0.109 | 0.064 | 0.107 | 0.070 |
| CMC-p | 0.049 | 0.053 | 0.051 | 0.064 | 0.043 |  | 0.051 | 0.052 | 0.049 | 0.045 | 0.052 |
| KBAC | 0.043 | 0.049 | 0.053 | 0.060 | 0.049 |  | 0.063 | 0.052 | 0.041 | 0.056 | 0.062 |
| KMR | 0.041 | 0.053 | 0.047 | 0.057 | 0.053 |  | 0.054 | 0.041 | 0.055 | 0.052 | 0.053 |
| C-alpha | 0.049 | 0.062 | 0.051 | 0.048 | 0.051 |  | 0.070 | 0.050 | 0.043 | 0.063 | 0.047 |
| WSS | 0.055 | 0.050 | 0.042 | 0.058 | 0.045 |  | 0.057 | 0.056 | 0.064 | 0.049 | 0.045 |
| ORWSS | 0.051 | 0.057 | 0.057 | 0.059 | 0.044 |  | 0.049 | 0.040 | 0.045 | 0.049 | 0.048 |
| VT | 0.051 | 0.054 | 0.057 | 0.064 | 0.058 |  | 0.054 | 0.050 | 0.044 | 0.062 | 0.039 |
| SKAT_1_ | 0.046 | 0.046 | 0.044 | 0.050 | 0.046 |  | 0.058 | 0.046 | 0.032 | 0.062 | 0.032 |
| SKAT_b_ | 0.050 | 0.052 | 0.054 | 0.034 | 0.056 |  | 0.066 | 0.046 | 0.056 | 0.054 | 0.050 |
| SKAT-C | 0.050 | 0.052 | 0.040 | 0.046 | 0.046 |  | 0.066 | 0.044 | 0.042 | 0.064 | 0.036 |
| SKAT-A | 0.050 | 0.052 | 0.038 | 0.040 | 0.046 |  | 0.066 | 0.044 | 0.032 | 0.066 | 0.038 |
| WSS-*t* | 0.049 | 0.051 | 0.054 | 0.061 | 0.038 |  | 0.054 | 0.051 | 0.047 | 0.065 | 0.043 |
| ORWSS-*t* | 0.047 | 0.052 | 0.065 | 0.064 | 0.049 |  | 0.046 | 0.046 | 0.054 | 0.050 | 0.041 |
| DWSS-*t* | 0.045 | 0.054 | 0.060 | 0.062 | 0.050 |  | 0.052 | 0.053 | 0.055 | 0.048 | 0.051 |
| DSS-*t* | 0.044 | 0.049 | 0.049 | 0.058 | 0.044 |  | 0.052 | 0.050 | 0.046 | 0.046 | 0.047 |
| VWSS-*t* | 0.049 | 0.051 | 0.056 | 0.061 | 0.058 |  | 0.053 | 0.048 | 0.046 | 0.063 | 0.049 |
| VORWSS-*t* | 0.047 | 0.052 | 0.064 | 0.064 | 0.049 |  | 0.045 | 0.047 | 0.057 | 0.051 | 0.044 |
| VDWSS-*t* | 0.046 | 0.052 | 0.061 | 0.061 | 0.051 |  | 0.052 | 0.052 | 0.056 | 0.055 | 0.052 |
| VDSS-*t* | 0.047 | 0.048 | 0.060 | 0.052 | 0.044 |  | 0.047 | 0.053 | 0.056 | 0.052 | 0.049 |
