# Supplementary material for: A Robust GWSS Method to Simultaneously Detect Rare and Common Variants for Complex Disease
Source: PLoS One. 2015 Apr 16;10(4):e0120873. doi: 10.1371/journal.pone.0120873 (PMC4399906; doi:10.1371/journal.pone.0120873)
Supplement: S2 Table — (DOC) [file pone.0120873.s003.doc]

**Table S2. Detection power for identical MAF distributions of signal and noise rare variants (other methods).**

|  |  | | | | | | | | | | |  |  | | | | | | | | | | |
| --- | --- | --- | --- | --- | --- | --- | --- | --- | --- | --- | --- | --- | --- | --- | --- | --- | --- | --- | --- | --- | --- | --- | --- |
|  | 1. *OR_j_*=2 (RVs), 1.5 (CV) | | | | |  | 1. *OR_j_*=1/2 (RVs), 1/1.5 (CV) | | | | |  | 1. *OR_j_*=2 (RVs), 1.5 (CV) | | | | |  | 1. *OR_j_*=1/2 (RVs), 1/1.5 (CV) | | | | |
| $n_{true}^{RV}$ | 8 | 8 | 8 | 7 | 7 |  | 8 | 8 | 8 | 7 | 7 |  | 8 | 8 | 8 | 7 | 7 |  | 8 | 8 | 8 | 7 | 7 |
| $n_{true}^{CV}$ | 0 | 0 | 0 | 1 | 1 |  | 0 | 0 | 0 | 1 | 1 |  | 0 | 0 | 0 | 1 | 1 |  | 0 | 0 | 0 | 1 | 1 |
| $n_{noise}^{RV}$ | 0 | 8 | 4 | 8 | 4 |  | 0 | 8 | 4 | 8 | 4 |  | 0 | 8 | 4 | 8 | 4 |  | 0 | 8 | 4 | 8 | 4 |
| $n_{noise}^{CV}$ | 0 | 0 | 4 | 0 | 4 |  | 0 | 0 | 4 | 0 | 4 |  | 0 | 0 | 4 | 0 | 4 |  | 0 | 0 | 4 | 0 | 4 |
| SSU | 0.96 | 0.94 | 0.35 | 0.90 | 0.64 |  | 0.94 | 0.90 | 0.27 | 0.87 | 0.46 |  | 1.00 | 0.99 | 0.76 | 0.99 | 0.91 |  | 0.79 | 0.79 | 0.27 | 0.74 | 0.39 |
| Sum Test | 1.00 | 0.95 | 0.62 | 0.98 | 0.77 |  | 0.62 | 0.44 | 0.19 | 0.18 | 0.10 |  | 1.00 | 0.99 | 0.95 | 1.00 | 0.97 |  | 0.42 | 0.35 | 0.26 | 0.20 | 0.16 |
| CMC-p | 0.97 | 0.78 | 0.82 | 0.86 | 0.88 |  | 0.66 | 0.48 | 0.47 | 0.67 | 0.62 |  | 0.99 | 0.85 | 0.87 | 0.94 | 0.94 |  | 0.51 | 0.39 | 0.42 | 0.53 | 0.52 |
| KBAC | 1.00 | 0.99 | 0.83 | 0.98 | 0.87 |  | 0.94 | 0.83 | 0.46 | 0.73 | 0.39 |  | 1.00 | 1.00 | 0.88 | 1.00 | 0.95 |  | 0.81 | 0.70 | 0.38 | 0.51 | 0.27 |
| KMR | 0.96 | 0.94 | 0.37 | 0.91 | 0.65 |  | 0.94 | 0.91 | 0.29 | 0.88 | 0.48 |  | 1.00 | 0.99 | 0.78 | 0.99 | 0.92 |  | 0.79 | 0.79 | 0.30 | 0.75 | 0.40 |
| C-alpha | 0.97 | 0.94 | 0.31 | 0.93 | 0.63 |  | 0.94 | 0.91 | 0.22 | 0.81 | 0.33 |  | 1.00 | 0.99 | 0.80 | 0.99 | 0.93 |  | 0.81 | 0.80 | 0.30 | 0.69 | 0.33 |
| WSS | 1.00 | 0.97 | 0.88 | 0.99 | 0.93 |  | 0.78 | 0.57 | 0.42 | 0.32 | 0.27 |  | 1.00 | 0.99 | 0.93 | 1.00 | 0.97 |  | 0.66 | 0.56 | 0.39 | 0.27 | 0.22 |
| ORWSS | 0.97 | 0.89 | 0.73 | 0.91 | 0.83 |  | 0.94 | 0.86 | 0.67 | 0.90 | 0.73 |  | 0.99 | 0.90 | 0.61 | 0.93 | 0.79 |  | 0.83 | 0.70 | 0.46 | 0.79 | 0.56 |
| VT | 0.99 | 0.92 | 0.97 | 0.93 | 0.95 |  | 0.77 | 0.59 | 0.68 | 0.63 | 0.73 |  | 1.00 | 0.99 | 1.00 | 1.00 | 0.99 |  | 0.57 | 0.50 | 0.59 | 0.51 | 0.61 |
| SKAT_1_ | 0.95 | 0.93 | 0.38 | 0.89 | 0.61 |  | 0.93 | 0.90 | 0.28 | 0.91 | 0.52 |  | 1.00 | 0.99 | 0.79 | 0.99 | 0.92 |  | 0.81 | 0.80 | 0.26 | 0.75 | 0.36 |
| WSS-*t* | 1.00 | 0.97 | 0.97 | 0.98 | 0.96 |  | 0.69 | 0.53 | 0.51 | 0.45 | 0.46 |  | 1.00 | 1.00 | 1.00 | 1.00 | 0.99 |  | 0.46 | 0.43 | 0.45 | 0.37 | 0.36 |
| DSS-*t* | 0.97 | 0.92 | 0.65 | 0.94 | 0.78 |  | 0.94 | 0.88 | 0.56 | 0.90 | 0.68 |  | 0.99 | 0.95 | 0.83 | 0.97 | 0.91 |  | 0.81 | 0.74 | 0.47 | 0.82 | 0.53 |
| VWSS-*t* | 0.99 | 0.92 | 0.96 | 0.92 | 0.95 |  | 0.75 | 0.58 | 0.66 | 0.60 | 0.71 |  | 1.00 | 0.99 | 0.99 | 0.99 | 0.99 |  | 0.50 | 0.44 | 0.55 | 0.48 | 0.58 |
