# Supplementary material for: A Robust GWSS Method to Simultaneously Detect Rare and Common Variants for Complex Disease
Source: PLoS One. 2015 Apr 16;10(4):e0120873. doi: 10.1371/journal.pone.0120873 (PMC4399906; doi:10.1371/journal.pone.0120873)
Supplement: S3 Table — (DOC) [file pone.0120873.s004.doc]

**Table S3. Type I error (significant level=0.05) and detection power (ρ=0) for different MAF distributions of signal and noise rare variants.**

|  | Type I error | | | |  | 1. *OR_j_*=2 (RVs), 1.5 (CV) | | | |  | 1. *OR_j_*=1/2 (RVs), 1/1.5 (CV) | | | |
| --- | --- | --- | --- | --- | --- | --- | --- | --- | --- | --- | --- | --- | --- | --- |
| $n_{true}^{RV}$ | 8 | 8 | 7 | 7 |  | 8 | 8 | 7 | 7 |  | 8 | 8 | 7 | 7 |
| $n_{true}^{CV}$ | 0 | 0 | 1 | 1 |  | 0 | 0 | 1 | 1 |  | 0 | 0 | 1 | 1 |
| $n_{noise}^{RV}$ | 8 | 4 | 8 | 4 |  | 8 | 4 | 8 | 4 |  | 8 | 4 | 8 | 4 |
| $n_{noise}^{CV}$ | 0 | 4 | 0 | 4 |  | 0 | 4 | 0 | 4 |  | 0 | 4 | 0 | 4 |
| SSU | 0.049 | 0.045 | 0.037 | 0.052 |  | 0.58 | 0.09 | 0.71 | 0.44 |  | 0.50 | 0.08 | 0.60 | 0.26 |
| SSU*w* | 0.035 | 0.039 | 0.032 | 0.043 |  | 0.59 | 0.56 | 0.72 | 0.72 |  | 0.47 | 0.49 | 0.64 | 0.60 |
| *w*SSU | 0.042 | 0.053 | 0.056 | 0.044 |  | 0.74 | 0.78 | 0.81 | 0.84 |  | 0.57 | 0.61 | 0.66 | 0.67 |
| Sum Test | 0.055 | 0.048 | 0.055 | 0.042 |  | 0.71 | 0.31 | 0.87 | 0.53 |  | 0.23 | 0.10 | 0.10 | 0.05 |
| CMC-p | 0.054 | 0.043 | 0.061 | 0.060 |  | 0.54 | 0.56 | 0.72 | 0.70 |  | 0.15 | 0.16 | 0.42 | 0.36 |
| KBAC | 0.038 | 0.054 | 0.067 | 0.043 |  | 0.83 | 0.44 | 0.92 | 0.68 |  | 0.52 | 0.21 | 0.35 | 0.16 |
| KMR | 0.053 | 0.048 | 0.038 | 0.055 |  | 0.59 | 0.10 | 0.72 | 0.45 |  | 0.51 | 0.08 | 0.61 | 0.27 |
| C-alpha | 0.064 | 0.050 | 0.044 | 0.056 |  | 0.60 | 0.09 | 0.78 | 0.49 |  | 0.53 | 0.08 | 0.52 | 0.17 |
| WSS | 0.045 | 0.058 | 0.041 | 0.051 |  | 0.80 | 0.58 | 0.92 | 0.76 |  | 0.37 | 0.24 | 0.13 | 0.12 |
| ORWSS | 0.067 | 0.063 | 0.058 | 0.041 |  | 0.53 | 0.35 | 0.75 | 0.59 |  | 0.52 | 0.30 | 0.68 | 0.46 |
| VT | 0.041 | 0.056 | 0.053 | 0.051 |  | 0.80 | 0.86 | 0.80 | 0.87 |  | 0.46 | 0.50 | 0.52 | 0.55 |
| SKAT_1_ | 0.062 | 0.038 | 0.038 | 0.058 |  | 0.65 | 0.09 | 0.75 | 0.44 |  | 0.51 | 0.09 | 0.58 | 0.26 |
| SKAT_b_ | 0.058 | 0.040 | 0.058 | 0.052 |  | 0.67 | 0.59 | 0.67 | 0.65 |  | 0.52 | 0.53 | 0.66 | 0.57 |
| SKAT-C | 0.040 | 0.048 | 0.042 | 0.050 |  | 0.67 | 0.46 | 0.80 | 0.72 |  | 0.84 | 0.74 | 0.93 | 0.90 |
| SKAT-A | 0.040 | 0.030 | 0.038 | 0.046 |  | 0.67 | 0.52 | 0.72 | 0.61 |  | 0.84 | 0.74 | 0.86 | 0.86 |
| WSS-*t* | 0.032 | 0.056 | 0.056 | 0.046 |  | 0.85 | 0.83 | 0.90 | 0.90 |  | 0.38 | 0.37 | 0.32 | 0.28 |
| ORWSS-*t* | 0.054 | 0.050 | 0.056 | 0.053 |  | 0.63 | 0.60 | 0.76 | 0.75 |  | 0.55 | 0.55 | 0.69 | 0.64 |
| DWSS-*t* | 0.044 | 0.050 | 0.053 | 0.052 |  | 0.70 | 0.67 | 0.80 | 0.80 |  | 0.57 | 0.57 | 0.71 | 0.64 |
| DSS-*t* | 0.051 | 0.048 | 0.047 | 0.062 |  | 0.59 | 0.26 | 0.78 | 0.54 |  | 0.50 | 0.21 | 0.66 | 0.41 |
| VWSS-*t* | 0.036 | 0.059 | 0.052 | 0.056 |  | 0.79 | 0.87 | 0.78 | 0.88 |  | 0.45 | 0.49 | 0.52 | 0.54 |
| VORWSS-*t* | 0.054 | 0.051 | 0.056 | 0.052 |  | 0.63 | 0.61 | 0.76 | 0.75 |  | 0.55 | 0.55 | 0.69 | 0.64 |
| VDWSS-*t* | 0.044 | 0.053 | 0.048 | 0.053 |  | 0.72 | 0.71 | 0.79 | 0.80 |  | 0.57 | 0.59 | 0.70 | 0.66 |
| VDSS-*t* | 0.051 | 0.053 | 0.054 | 0.059 |  | 0.67 | 0.67 | 0.76 | 0.73 |  | 0.56 | 0.57 | 0.64 | 0.64 |
