# Supplementary material for: A Robust GWSS Method to Simultaneously Detect Rare and Common Variants for Complex Disease
Source: PLoS One. 2015 Apr 16;10(4):e0120873. doi: 10.1371/journal.pone.0120873 (PMC4399906; doi:10.1371/journal.pone.0120873)
Supplement: S4 Table — (DOC) [file pone.0120873.s005.doc]

**Table S4. Robustness of all methods in situations of identical/different MAF distributions of signal and noise rare variants (other methods).**

|  | 1. One factor | | |  | 1. Two factors | | |
| --- | --- | --- | --- | --- | --- | --- | --- |
|  | Direction | Noise RVs | Noise CVs |  | Noise RVs  X  Direction | Noise CVs  X  Direction | signal CVs  X  Direction |
| SSU | X | X | ⚫ |  | X | X (∆) | X |
| Sum Test | ⚫ | X | ⚫ |  | ⚫ | ∆ | ⚫ |
| CMC-p | ⚫ | ⚫ | X |  | X | X | ⚫ |
| KBAC | ∆ | X | ⚫ |  | ⚫ | ⚫ (∆) | ∆ (⚫) |
| KMR | X | X | ⚫ |  | X | X | X |
| C-alpha | X | X | ⚫ |  | X | ∆ | ∆ (⚫) |
| WSS | ⚫ | X | ∆ |  | ⚫ | ∆ | ⚫ |
| ORWSS | X (⚫) | ∆ | ⚫ |  | X | X (⚫) | X (⚫) |
| VT | ⚫ | ∆ | X |  | ⚫ | X | X |
| SKAT_1_ | X (⚫) | X (⚫) | ⚫ |  | X | ⚫ | X |
| WSS-*t* | ⚫ | X | X |  | ⚫ | X | ∆ |
| DSS-*t* | X | X | ⚫ |  | X | X | X |
| VWSS-*t* | ⚫ | ∆ | X |  | ⚫ | X | X |
| Abbreviation: RVs, rare variants; CVs, common variants; OR, odds ratio.  ^a^ The symbol inside brackets represents the sensitivity under the situation of different MAF distributions, if it is different from that of identical MAF distribution. ^b^ The symbol ⚫: sensitive; ∆: slightly sensitive; and X: non-sensitive. | | | | | | | |
