# Supplementary material for: A Robust GWSS Method to Simultaneously Detect Rare and Common Variants for Complex Disease
Source: PLoS One. 2015 Apr 16;10(4):e0120873. doi: 10.1371/journal.pone.0120873 (PMC4399906; doi:10.1371/journal.pone.0120873)
Supplement: S5 Table — (DOC) [file pone.0120873.s006.doc]

**Table S5. Effects of disease liability using the Genetic Analysis Workshop 17 data (other methods and details of the GWSS method).**

| Chromosome | 1 | 1 | 2 | 3 | 3 | 4 | 9 | 10 | 11 | 13 | 14 | 18 | 19 | 22 |
| --- | --- | --- | --- | --- | --- | --- | --- | --- | --- | --- | --- | --- | --- | --- |
| Gene symbol | *ADAM15* | *MSH4* | *ARL6IP2* | *EPHB1* | *TRIM42* | *FAM13A1* | *SHC3* | *FRMPD2* | *DGKZ* | *FLT1* | *NFKBIA* | *MBD1* | *GDF15* | *SUSD2* |
| no. of RVs | 22 | 16 | 9 | 6 | 33 | 27 | 4 | 42 | 17 | 25 | 6 | 10 | 4 | 36 |
| no. of CVs | 8 | 4 | 4 | 2 | 6 | 7 | 1 | 8 | 5 | 10 | 2 | 2 | 6 | 9 |
| ***p*-value^a^:** |  |  |  |  |  |  |  |  |  |  |  |  |  |  |
| WSS | **0.001** | 0.006 | 0.007 | 0.023 | 0.080 | 0.005 | **0.001** | **0.001** | **0.001** | 0.002 | 0.009 | 0.331 | 0.016 | **0.001** |
| ORWSS | 0.002 | 0.003 | 0.034 | **0.001** | 0.043 | 0.010 | **0.001** | 0.002 | 0.006 | 0.002 | 0.018 | 0.007 | 0.002 | 0.016 |
| VT | 0.221 | 0.263 | 0.071 | 0.156 | **0.001** | 0.122 | 0.159 | 0.115 | **0.001** | 0.009 | 0.003 | 0.013 | **0.001** | **0.001** |
| SKAT_1_ | **<.0001** | **<.0001** | 0.034 | **<.0001** | 0.601 | 0.314 | **0.001** | 0.001 | 0.003 | 0.108 | 0.027 | 0.022 | 0.052 | 0.022 |
| Abbreviation: RVs, rare variants; CVs, common variants.  ^a^ The detection *p*-value was calculated from 1000 permutations; bold indicates *p*-value reaches the significance level (i.e., ≤0.001). | | | | | | | | | | | | | | |
